# Advances in Taxonomy, Ecology, and Biogeography of Dirivultidae (Copepoda) Associated with Chemosynthetic Environments in the Deep Sea

**DOI:** 10.1371/journal.pone.0009801

**Published:** 2010-08-31

**Authors:** Sabine Gollner, Viatcheslav N. Ivanenko, Pedro Martínez Arbizu, Monika Bright

**Affiliations:** 1 Department of Marine Biology, University of Vienna, Vienna, Austria; 2 Department of Invertebrate Zoology, Moscow State University, Moscow, Russia; 3 Deutsches Zentrum für Marine Biodiversitätsforschung, Forschungsinstitut Senckenberg, Wilhelmshaven, Germany; Paleontological Institute, Russian Federation

## Abstract

**Background:**

Copepoda is one of the most prominent higher taxa with almost 80 described species at deep-sea hydrothermal vents. The unique copepod family Dirivultidae with currently 50 described species is the most species rich invertebrate family at hydrothermal vents.

**Methodology/Principal Findings:**

We reviewed the literature of Dirivultidae and provide a complete key to species, and map geographical and habitat specific distribution. In addition we discuss the ecology and origin of this family.

**Conclusions/Significance:**

Dirivultidae are only present at deep-sea hydrothermal vents and along the axial summit trough of midocean ridges, with the exception of *Dirivultus dentaneus* found associated with *Lamellibrachia* species at 1125 m depth off southern California. To our current knowledge Dirivultidae are unknown from shallow-water vents, seeps, whale falls, and wood falls. They are a prominent part of all communities at vents and in certain habitat types (like sulfide chimneys colonized by pompei worms) they are the most abundant animals. They are free-living on hard substrate, mostly found in aggregations of various foundation species (e.g. alvinellids, vestimentiferans, and bivalves). Most dirivultid species colonize more than one habitat type. Dirivultids have a world-wide distribution, but most genera and species are endemic to a single biogeographic region. Their origin is unclear yet, but immigration from other deep-sea chemosynthetic habitats (stepping stone hypothesis) or from the deep-sea sediments seems unlikely, since Dirivultidae are unknown from these environments. Dirivultidae is the most species rich family and thus can be considered the most successful taxon at deep-sea vents.

## Introduction

Copepoda are estimated to contribute more than 15% to the total number of animal species known from deep-sea hydrothermal vents worldwide [1]. Almost 80 species are currently described from the orders Harpacticoida, Calanoida, Cyclopoida, Poecilostomatoida and Siphonostomatoida, but many more species await identification and description [2]. The Dirivultidae, a family belonging to the Siphonostomatoida, is the most diverse one of all animal families at vents with 13 genera including 50 described species. The most diverse genus is *Stygiopontius* with 21 species. Similar diverse faunal groups at deep-sea hydrothermal vents are Gastropoda with about 100 described species, including the prominent family Lepetodrilidae with 20 species and within this family the large genus *Lepetodrilus* (13 known species). Polychaeta are also represented with currently 111 species and the Polynoidae including 24 species [3].

In hard substrate ecosystems like many hydrothermal vents, copepods can be the most abundant and diverse meiofaunal taxon [4], [5]. Copepods in general play an important role in various ecosystems, being usually the second dominant higher meiofauna taxon following the nematodes [6]. They are known from marine and freshwater plankton, marine sediments, cryptic habitats (soil, forest litter, terrestrial mosses, tree holes), subterranean habitats (springs, pools in caves), anchialine caves, deep-sea vents, and as animal and plant associates [7]. Their ecological importance is high and in some ecosystems as e.g. in the plankton, copepods are the main primary consumers. Copepods are essential for nutrient recycling and their fecal pellets are a central source for detritus feeders, but also the animals themselves are an abundant feeding source for macrofauna [7].

Dirivultidae are found in frequent and diverse numbers at hydrothermal vents around the globe. For this review we developed a simple identification table which should help scientists to identify these copepods easy in future. Ecological aspects such as abundance and diversity patterns are evaluated. We also provide an update on current distribution patterns of this unique family and discuss the origin of Dirivultidae.

## Methods

We reviewed the literature of Dirivultidae, including all species descriptions and ecological studies. Original species descriptions were used to develop a key to genera and species. We investigated the occurrence of dirivultids in chemosynthetic habitats such as hydrothermal vents, cold seeps, wood falls and whale falls in the deep sea to provide a complete overview of the distribution of this unique family. In addition, we also considered trophic interactions and compared abundance and diversity patterns of Dirivultidae in various ecosystems and habitat types to gain insight into the ecology of these copepods. Biogeographical patterns were analyzed by separation into four large regions: the Atlantic, North East Pacific, East Pacific, and West Pacific, following the definition of Desbruyères et al. [3]. We use the thus obtained information to discuss the origin of the Dirivultidae.

## Results and Discussion

### Taxonomy

**Figure 1:**
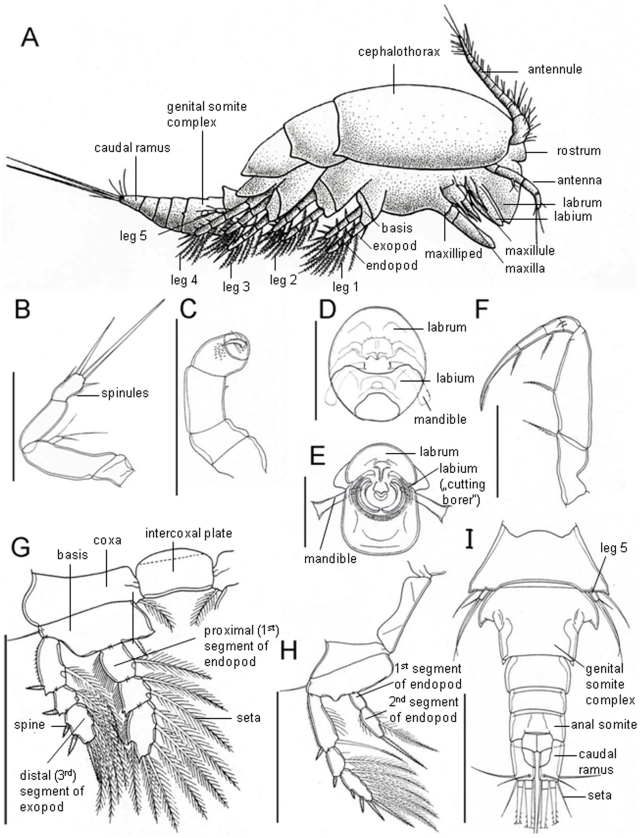
Schematic drawings of dirivultid morphology and important characters for identification on genus and species level. The figure was created by selecting drawings of previous publications and adding additional information to illustrate the key to genera (Table 1) and to species (Tables 2, 3, 4). A: lateral view of a dirivultid (length ∼1 mm) [3]. B–H ventral view of: B: antenna of *Stygiopontius lauensis* [18]. C: antenna of *Ceuthoecetes introversus* [9]. D: oral cone of *Benthoxynus spiculifer* [13]. E: oral cone of *C. introversus* [9]. F: maxilliped of *S. lauensis* [18]. G: leg 1 of *S. lauensis* [18]. H: leg 4 of *S. lauensis* [18]. I: dorsal view of urosome of *Aphotopontius acanthinus* [19]. Scale bars: B–H: 100 µm; I: 200 µm.

**Figure 2:**
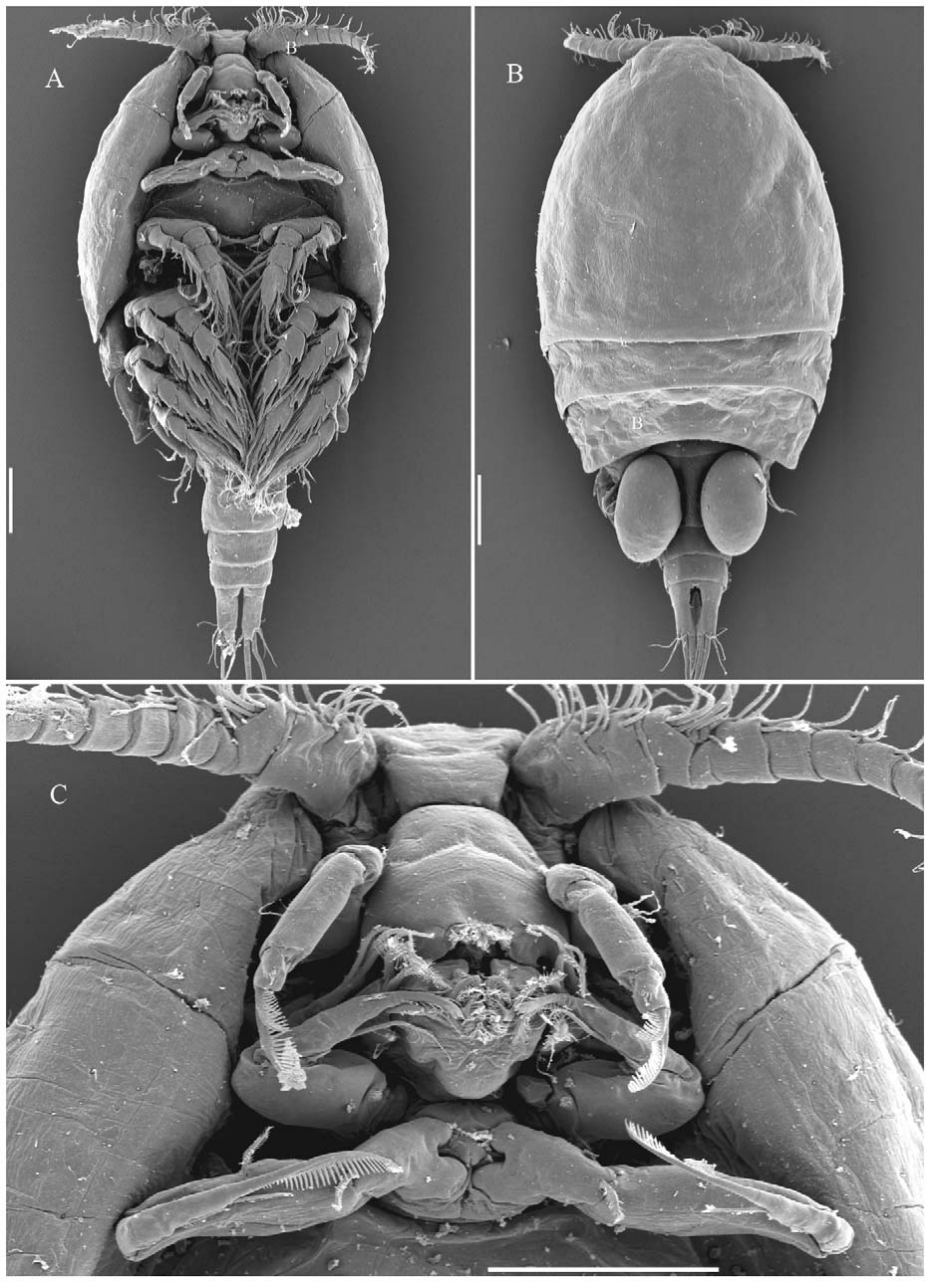
*Stygiopontius pectinatus* (female) SEM micrographs. A: habitus, ventral view. B: habitus, dorsal view. C: oral cone and anterior appendages. Scale bars 100 µm. (A, B: [3]; C: by VNI).

Dirivultidae belong to the siphonostomatoid copepods and their morphological characteristics include: The body is cyclopiform with length ranging from 0.5 to 1.8 mm (Figure 1A, Figure 2). The prosome is 4 segmented, the urosome 4–5 segmented in females and 5–6 segmented in males. The first urosomite bears the leg 5. The oral cone is short and robust formed by labrum and labium (Figure 1D). In addition to the oral cone in the genera *Ceuthoecetes*, *Dirivultus* and *Nilva* a cutting borer is formed by the labium (Figure 1E). Mandible, maxillule, maxilla, and maxilliped are present (Figure 1A, 1D, 1E). Rami of legs 1 to 3 and exopod of leg 4 are 3-segmented (Figure 1G). Endopod of leg 4 is 2-segmented (Figure 1 H). The development is as follows: females carry two egg-sacks each containing one, frequently two (rarely more) large, yolky eggs; nauplii hatch as non-feeding lecithotrophic larvae, lacking mouth and labrum, and lacking a naupliar arthrite on the coxa of the antenna [8]. The exact number of naupliar stages is unknown; the lecithotrophic nauplius may moult directly into the first copepodid stage. Five copepodid stages with well developed mouth parts and gut follow, the sixth stage being the adult.

The presumably derived characters distinguishing Dirivultidae from Ecbathyriontidae and other siphonostomatoids are the 2-segmented endopod of leg 4 (is 3-segmented in Ecbathyriontidae and many other siphonostomatoids) and the fusion of ancestral segments 3–8 in the proximal part of the antennule into one compound segment which is armed with 6 pairs of setae. Morphological observations suggest that Ecbathyriontidae, a family consisting of a single species (a new species and genus is in preparation, pers. com. VNI) (*Ecbathyrion prolixicauda*, Humes 1987) and found at hydrothermal vents, can be considered the only sister-group of Dirivultidae [9]. The synapomorphy of the taxon Ecbathyriontidae – Dirivultidae is the presence of a double segment in the female antennule. This double segment is armed with 2 pairs of setae and formed by fusion of two segments which correspond to the ancestral segments 15 and 16 [10].

The type genus of the Dirivultidae is *Dirivultus* Humes & Dojiri, 1980, and the other 12 genera are *Aphotopontius* Humes, 1987; *Benthoxynus* Humes, 1984; *Ceuthoecetes* Humes & Dojiri, 1980; *Chasmatopontius* Humes, 1990; *Exrima* Humes, 1987; *Fissuricola* Humes, 1987; *Humesipontius* Ivanenko & Ferrari, 2003; *Nilva* Humes, 1987; *Rhogobius* Humes, 1987; *Rimipontius* Humes, 1996; *Scotoecetes* Humes, 1987; and *Stygiopontius* Humes, 1987 (Table 1). The genera can be mainly distinguished by the setation of the endopod of leg 4 (Figure 1H). The genera *Chasmatopontius* and *Fissuricola* are considered as basal due to the existence of 3 inner setae on the distal (second) endopodal segment of leg 4. These 3 setae indicate that the ancestor had three endopodal segments on leg 4. The distal (third) and middle (second) segments of a 3-segmented condition are fused into a distal double-segment in the 2-segmented condition. The presence of this former middle segment on the endopod of leg 4 is evidenced by the retention of 1 inner proximal seta of this segment (2 setae are indicated for the ancestor of siphonostomatoid). Ten other genera of dirivultids are characterized by a remarkably uniform 2-segmented endopod of leg 4. The distal endopodal segment of the 2-segmented endopod is armed with 2 setae at most, 1 terminal and 1 inner. The inner seta is lost in several genera of dirivultids. The proximal endopodal segment of leg 4 in dirivultids is armed with 1 inner seta at most as in the ancestral state of siphonostomatoids (this seta is lost in several genera of dirivultids). The endopod of leg 4 is lost completely in the monotypic genus *Humesipontius*. Table 1 is a key to genera featuring setation and some additional characters allowing genus identification. Schematic drawings of dirivultid morphology and important characters for identification are given in Figure 1.

**Table 1:** Genus key of Dirivultidae.

genus	#	1st	2nd	comment
*Benthoxynus*	2	0-0	0,I,0	leg 3 exopod, 1^st^ segment without setae (in contrast to *Exrima*, *Rimipontius*)
*Exrima*	2	0-0	0,I,0	leg 3 exopod, 1^st^ segment with setae (in contrast to *Benthoxynus*)
*Rimipontius*	1	0-0	0,I,0	caudal ramus with 5 setae (on contrast to 6 in other Dirivultidae)
*Ceuthoecetes*	4	0-0	0,I,1	A2 has hooklike claw, A1 10 segmented (in male and female)
*Dirivultus*	2	0-0	0,I,1	A2 has hooklike claw, A1 13 segmented (female) or 12 seg (male), oral cone with spines
*Nilva*	1	0-0	0,I,1	A2 has hooklike claw, urosome with dorsal hump
*Stygiopontius*	21	0-0	0,I,1	leg 1 endopod is 3 segmented in female and male, leg 5 male normally developed (1 segment)
*Scotoecetes*	1	0-0	0,I,1	leg 1 endopod is 2 segmented in female, leg 5 in male reduced to small ridge with 3 setae
*Chasmatopontius*	1	0-0	0,0,3	urosome 4 segmented in female, 5 segmented in male (in contrast to other Dirivultidae)
*Aphotopontius*	10	0-1	0,I,1	no lobes at anal somite
*Rhogobius*	3	0-1	0,I,1	2 lobes at anal somite (in contrast to *Aphotopontius*)
*Fissuricola*	1	0-1	0,I,3	
*Humesipontius*	1	absent	absent	

Dirivultid genera and number of described species within each genus (#). Genera can be distinguished by the number of setae (Arabic numbers) and spines (Latin numbers) on their leg 4 endopod. 1^st^ indicates setation of the first segment of leg 4 endopod, 2^nd^ indicates setation of the second segment of leg 4 endopod (see for example Figure 1H, showing leg 4 of *Stygiopontius*). The first step of genus identification is to analyze leg 4 endopod, afterwards the description of other characters should be followed. Other characters include number of segments and setation of other legs (terminology of different parts of legs see Figure 1A and 1G), number of setae on caudal rami and lobe presence/absence at anal somite (see for example Figure 1I showing *Aphotopontius* with 6 setae on caudal rami and anal somite without lobes), shape of antenna (A2) (Figure 1B shows a typical antenna of Dirivultidae; Figure 1C shows the antenna with a hook like claw as typical for the genera *Ceuthoecetes*, *Dirivultus*, and *Nilva*), and number of segments in antennule (A1).

Fifty species belong to the 13 dirivultid genera (Table 2, Table 3, and Table 4) [11]–[24]. Six genera (*Chasmatopontius*, *Fissuricola*, *Humesipontius*, *Rimipontius*, *Nilva*, and *Scotoecetes*) are monotypic; 3 genera (*Benthoxynus*, *Dirivultus*, *Exrima*) contain 2 species; *Rhogobius* holds 3 species and *Ceuthoecetes* 4 species. Most diverse genera are *Aphotopontius* and *Stygiopontius* with 10 and 21 species, respectively. In addition, our collection contains 2 species of *Stygiopontius* and 1 species of *Chasmatopontius* which are new to science but undescribed yet (SG, VNI pers. obs.). Tables 2 to [Table pone-0009801-t003] 4 provide keys of genus specific characters allowing species identification within genera. Synonyms are as followed: *Aphotopontius rapunculus* (Humes and Segonzac, 1998) was transferred to *Rhogobius rapunculus* (Humes, 1987) [2; IVN in prep.]; *A. temperatus* (Humes, 1997) was synonymized with *A. atlanteus* [2]. *Stygiopontius lumiger* (Humes, 1989) and *S. bulbisetiger* (Humes, 1996) were synonymized with *S. sentifer* and *S. pectinatus*, respectively [2].

**Table 2:** Species key of Dirivultidae: *Aphotopontius*.

*Aphotopontius*	caudal ramus l∶w	f genital somite	m genital somite	other characters
*A. baculigerus*	14∶1 (f) 9∶1 (m)	with small S	no info	rostrum rounded, anal segment smooth
*A. limatulus*	10∶1 (f) 8∶1 (m)	no S, rounded	no S	rostrum straight, anal segment with spinules
*A. forcipatus*	5∶1	no S, rounded	no S	broad genital somite, male leg 6 with 2 setae
*A. arcuatus*	5∶1, concave!	with S	no S	mxp slender, male leg 6 with 1 seta,
				spinules at outer margin of caudal ramus
*A. flexispina*	4∶1	no S, rounded	male unknown	mxp: spine on second segment has curved tip,
				claw is pectinate
*A. probolus*	3∶1	with S	no S	prominent process between mxp and leg 1,
				spinules at outer margin of caudal ramus
*A. acanthinus*	2∶1	with S	with S	basis leg 1 mammiliform, m A1 5^th^ seg with 1 spine
*A. mammillatus*	2∶1	hourglass shaped	with S	basis leg 1 mammiliform, m A1 5^th^ seg with 2 spines
*A. hydronauticus*	2∶1	with S	male unknown	basis leg 1 rounded, mxp process not over leg 1
*A. atlanteus*	2∶1	no S	with S	basis leg 1 rounded, body broader than A. *hydronauticus*

First, species of this genus can be distinguished by the ratio of length to width of the caudal ramus (caudal ramus l∶w). Second, species can be discriminated by the presence or absence of spiniform processes (S) on the genital somite of female (f) and male (m) (e.g. Figure 1I shows *A. acanthinus* with a caudal ramus ratio of length∶width with 2∶1; the female genital double somite has spiniform processes). Other species characters include the shape of various parts of the body (i.e. the maxilliped (mxp), see Figure 1F).

**Table 3 pone-0009801-t003:** Species key of Dirivultidae: *Benthoxynus*, *Ceuthoecetes*, *Dirivultus*, *Exrima*, *Rhogobius*.

***Benthoxynus***	**characters**
*B. tumidiseta*	A1 f 11-segmented (m unknown), caudal ramus l∶w 7∶1
*B. spiculifer*	A1 f 18-segmented, A1 m 11 segmented), caudal ramus l∶w 5∶1
***Ceuthoecetes***	**characters**
*C. introversus*	leg 1, exopod 3^rd^ segment with inward spine (in contrast to other C), maxilla length 1^st^ to 2^nd^ segment 1∶1
*C. acanthothrix*	maxilla length 1^st^ to 2^nd^ segment 1∶1, spine on 2^nd^ seg of leg 3 exopod much longer than segment (other C∼same lenght)
*C. cristatus*	maxilla length 1^st^ to 2^nd^ segment 1∶1.5
*C. aliger*	mxp slender (in contrast to very a broad one in other C.), maxilla length 1^st^ to 2^nd^ segment 1∶1.5
***Dirivultus***	**characters**
*D. spinigulatus*	prosome has triangular shape, oral cone with 4 prominent posteroventral spines
*D. dentaneus*	prosome has rectangular shape, oral cone with 2 prominent posteroventral spines
***Exrima***	**characters**
*E. dolichopus*	length ratio caudal rami: last urosomite 1∶2, f genital segment triangle shape
*E. singula*	length ratio caudal rami: last urosomite 1∶2, f genital segment rectangular shape
***Rhogobius***	**characters**
*R. contractus*	genital segments equally developed, leg 5 2-segmented
*R. pressulus*	genital segment broad with 2 posterolateral processes, very small segment after genital somite, leg 5 2-segmented
*R. rapunculus*	leg 5 1-segmented

Used abbreviations: antennule (A1), female (f), male (m), length (l), width (w), maxilliped (mxp), segment (seg).

**Table 4:** Species key of Dirivultidae: *Stygiopontius*.

*Stygiopontius*	exo 4, 3^rd^	coxal setae	other characters
*S. appositus*	III, I, 4	none	peg-like structure on cephalothorax
*S. paxillifer*	III, I, 4	none	peg-like structure on cephalothorax, shorter claw on mxp (contrast to *S. appositus*)
*S. quadrispinosus*	III, I, 4	none	leg 3 endopod 3^rd^ segment with setation 1, 1, 3 (other S. 1, I, 3),
			extremely short innermost terminal seta at caudal ramus
*S. regius*	III, I, 4	none	leg 2 endopod 3^rd^ segment with setation 1, 1, 3 (other S. 1, 2, 3),
			m with broad genital segment, f with large leg 5 (looks like a flap)
*S. serratus*	III, I, 4	leg 2	A1 serrate, leg 1 intercoxal plate with 2 little knobs
*S. stabilitus*	III, I, 4	leg 2	A1 smooth, leg 1 intercoxal plate smooth
*S. latulus*	III, I, 4	leg 2	A1 smooth with large spine on 4^th^ segment, very broad body
*S. cladarus*	III, I, 4	leg 2, 3	(no spiniform processes at genital segment)
*S. brevispina*	III, I, 4	leg 1, 2	2 short spine-like setae on end of A2, short claw on mxp
*S. sentifer*	III, I, 4	leg 1, 2	maxilliped with very large thorn
*S. flexus*	III, I, 4	leg 1, 2	leg 3 endopod 3^rd^ segment with setation 1, 1, 3 (other S. 1, I, 3)
			1 pair postlateral spiniform processes at genital segment
*S. hispidulus*	III, I, 4	leg 1, 2	caudal rami smooth, leg 1 basis with spinules
*S. lauensis*	III, I, 4	leg 1, 2	caudal rami with spinules, leg 1 basis smooth
*S. mirus*	III, I, 4	leg 1	mxp with elongated 1^st^ segment
*S. pectinatus*	III, I, 4	leg 1, 2, 3	A2 claw like, mxp pectinate
*S. verruculatus*	II, 1, 4	none	knob on mxp, large genital segment
*S. rimivagus*	II, 1, 4	leg 1	
*S. cinctiger*	II, 1, 4	leg 1, 2	2^nd^ postgenital segment extremely short
*S. lomonosovi*	II, 1, 4	leg 1, 2	broader cephalothorax in contrast to *S. teres*
*S. teres*	II, 1, 4	leg 1, 2	more narrow cephalothorax in contrast to *S. lomonosovi*
*S. mucroniferus*	II, 1, 4	leg 2	mxp with spines (instead of setae in other S.) on 1^st^ and 2^nd^ segment

First, *Stygiopontius* species can be distinguished by the setation of the 3^rd^ exopodal segment of leg 4 (exo 4, 3^rd^) (setae are represented by Arabic numbers, spines by Latin numbers). Second, the number of coxal setae (if present, and on which leg it is present) has to be determined (see Figure 1G as an example of a coxal seta). Third, there are some additional characters allowing the final species identification of *Stygiopontius*. Used abbreviations: antennule (A1), antenna (A2), female (f), male (m), maxilliped (mxp).

Interestingly, only females or males are known in certain species despite the collection of sometimes thousands of specimens in a sample (see Table 5). For example, only females of *Stygiopontius pectinatus*, a species associated with the shrimp *Rimicaris exoculata* were found after inspection of more than 7400 individuals [20]. Whether the lack of finding both sexes has a biological background (e.g. parthenogenesis) or is simply due to wrong classification because of an acute sexual dimorphism remains to be studied, for example by life mating observations or by using genetic tools. Indeed, COI analyses of *Stygiopontius hispidulus* helped to find the male of that species (SG in prep.).

**Table 5:** Information on all dirivultid species including authorship, known sexes, biogeography, and habitat preference.

Species	authors	sex	A	NEP	EP	WP	biv	ves	alv	shr	bac	pla	ref #
*Aphotopontius acanthinus*	Humes & Lutz 1994	m, f			x			x			x		[25]
*Aphotopontius arcuatus*	Humes 1987	m, f			x		x	x	x				[16], [22], [25], [29], [30]
*Aphotopontius baculigerus*	Humes 1987	m, f			x								[16]
*Aphotopontius flexispina*	Humes 1987	f			x		x	x					[4], [30]
*Aphotopontius forcipatus*	Humes 1987	m, f	x	x			x	x		x			[5], [20], [29], [30]
*Aphotopontius hydronauticus*	Humes 1989	f			x			x					[4]
*Aphotopontius limatulus*	Humes 1987	m, f			x		x						[16], [22], [29], [30]
*Aphotopontius mammillatus*	Humes 1987	m, f			x		x	x					[4], [16], [22], [27], [29]
*Aphotopontius probolus*	Humes 1990	m, f			x		x	x					[4]
*Aphotopontius atlanteus*	Humes 1996	m, f	x				x						[22], [29]
*Benthoxynus spiculifer*	Humes 1984	m, f		x				x	x				[5], [16]
*Benthoxynus tumidiseta*	Humes 1989	f			x			x					[4]
*Ceuthoecetes acanthothrix*	Humes 1987	m			x		x	x					[4], [16], [22], [29], [30]
*Ceuthoecetes aliger*	Humes & Dojiri 1980	F			x		x	x					[4], [16], [22], [29], [30]
*Ceuthoecetes cristatus*	Humes 1987	m			x		x	x					[16], [30]
*Ceuthoecetes introversus*	Humes 1987	m			x		x	x					[4], [25]
*Chasmatopontius thescalus*	Humes 1990	m, f				x			x				[18]
*Dirivultus dentaneus*	Humes & Dojiri 1980	m, f						x					
*Dirivultus spinigulatus*	Humes 1999	m, f				x		x					
*Exrima dolichopus*	Humes 1987	f			x		x						[29], [30]
*Exrima singula*	Humes 1987	f			x		x	x					
*Fissuricola caritus*	Humes 1987	f			x								
*Humesipontius arthuri*	Ivanenko & Ferrari 2002	f		x				x					
*Nilva torifera*	Humes 1987	m, f			x		x	x					[16], [29], [30]
*Rhogobius contractus*	Humes 1987	m, f			x		x						[15], [16], [29], [30]
*Rhogobius pressulus*	Humes 1989	f			x								[16]
*Rhogobius rapunculus*	Humes & Segonzac 1998	f			x		x	x					[4], [29]
*Rimipontius mediospinifer*	Humes 1996	m, f	x				x			x		x	[22], [24], [28], [29]
*Scotoecetes introrsus*	Humes 1987	m, f			x		x	x	x				[30]
*Stygiopontius appositus*	Humes 1989	m			x								
*Stygiopontius brevispina*	Humes 1991	m, f				x							
*Stygiopontius cinctiger*	Humes 1987	f			x								[22], [30]
*Stygiopontius cladarus*	Humes 1996	m, f	x							x		x	[24], [28]
*Stygiopontius flexus*	Humes 1987	f			x		x	x			x		[4], [22], [25]
*Stygiopontius hispidulus*	Humes 1987	f			x		x	x	x				[4], [22], [30]
*Stygiopontius latulus*	Humes 1996	m	x							x			
*Stygiopontius lauensis*	Humes 1991	m, f				x							
*Stygiopontius lomonosovi*	Ivanenko et al. 2006	m, f	x				x						
*Stygiopontius mirus*	Humes 1996	m	x		x					x			[22]
*Stygiopontius mucroniferus*	Humes 1987	f			x			x		x			[4], [22]
*Stygiopontius paxillifer*	Humes 1989	m			x		x		x				[22], [25]
*Stygiopontius pectinatus*	Humes 1987	f	x			x			x	x		x	[5], [20], [24], [28]
*Stygiopontius quadrospinosus*	Humes 1987	m, f		x				x	x				[5], [16]
*Stygiopontius regius*	Humes 1996	m, f	x							x			
*Stygiopontius rimivagus*	Humes 1997	m	x		x		x						[22]
*Stygiopontius sentifer*	Humes 1987	f			x		x		x				[22], [29], [30]
*Stygiopontius serratus*	Humes 1996	m, f	x							x			
*Stygiopontius stabilitus*	Humes 1990	f			x	x		x	x				[4], [30]
*Stygiopontius teres*	Humes 1996	f	x							x			
*Stygiopontius verruculatus*	Humes 1987	m			x								[14], [22]

Sex is given for male (m) and female (f). We distinguished between four biogeographic regions: Atlantic (A), East Pacific (EP), North East Pacific (NEP), and West Pacific (WP). Habitat preferences were differentiated into bivalves (biv), vestimentiferans (ves), alvinellids (alv), shrimp (shr), bacterial mats (bac), and plankton (pla). X indicates presence. In addition to the authors' information, also other references are given for findings of each species (ref#).

### Ecology

#### Occurrence

Dirivultidae occur at deep-sea vents but have not been found in other chemosynthetic habitats such as shallow vents, seeps, whale falls (see Table 6) or wood falls nor in deep-sea or shallow-water sediments (PMA pers. obs.). At vents, however, they are not restricted to areas with vent flow, but can also survive away from vents on the bare basalt along the axial summit trough. Several species were encountered about 10 meters away from vents in the axial summit trough at the 9°50′N East Pacific Rise (EPR) region [25]. Also *Aphotopontius acanthinus* and *Stygiopontius hispidulus* were recently detected in samples taken about 1 km off-axis in the 9°50′N EPR region (SG pers. obs.).

**Table 6:** Relative abundance of dirivultid and harpacticoid copepods in chemosynthetic environments.

Location	depth (m)	habitat	Copepoda (% of meio)	Dirivultidae (% of cope)	Harpacticoida (% of cope)	ref #
**Seep infauna**						
Denmark	10 to 12	reduced sediments	no info	no info	no info	[41]
Santa Barbara	15	bac mats	0–1%	0	100	[42]
Santa Barbara	18	bac mats	7–14%	0	100	[43]
Santa Barbara	18	bac mats	2%	0	100	[44]
Santa Barbara	19	bac mats	6%	no info	no info	[46]
Gulf of Mexico	72	bac mats	0–46%	0	100	[47]
Gulf of Mexico	72	bac mats	1–16%	no info	no info	[48]
Black Sea	182–252	bac mats	0–59%	no info	no info	[49]
Norwegian margin	733	*Sclerolinum*	5%	0	100	[54]
	733	reduced sediments	12%	0	100	
Norwegian margin	746	*Sclerolinum*	3%	0	100	[54]
off Oregon	800	bac mats	0–1%	no info	no info	[52]
	800	under clams	0–4%	no info	no info	
Sagami Bay	1100–1200	under calms	1–13%	no info	no info	[50]
Blake Ridge	2154–2158	bac mats	0–54%	0	100	[55]
	2155–2157	under mussels	33–39%	0	100	
	2157	under xenophyophore	63–74%	0	100	
Gulf of Mexico	692–2238	bac mats	19–37%	0	100	[55]
Barents Sea	1255	bac mats	5%	0	100	[54]
Barents Sea	1286	sediment center	95%	0	100	[53]
	1288	*Sclerolinum*	7%	0	100	
	1287	bac mats	2%	0	100	
Barents Sea	1288	bac mats, *Sclerolinum*	8%	0	100	[51]
Barbados Trench	5000	sediment center	0%	no info	no info	[45]
	5000	under clams	1%	no info	no info	
	5000	near clams	2–3%	no info	no info	
**Seep epifauna**						
Gulf of Mexico	1400–2800	ass. vestimentifera	10–43%	absent (po SG)	majority (po, SG)	[40]
	1400–2800	ass. mussels	17–99%	absent (po SG)	majority (po, SG)	
**Vent infauna**						
Indonesia	3	reduced sediments	40–70%	no info	no info	[60]
New Zealand	8 to 11	bac mats	no info	no info	present	[56]
Papua New Guinea	0 to 27	bac mats	12–29%	0	12–29%	[57]
Mediterranean Sea	5 to 10	bac mats	no info	0	100	[58]
Guaymas	2000	bac mats	13%	no info	present	[38]
North Fiji Basin	2000	mussel sediment	0–3%	0	0–3%	[59]
**Vent epifauna**						
Guaymas	2000	ass. diverse fauna	60%	99%	a few	[38]
Juan de Fuca Ridge	2300	ass. *Paralvinella*	no info	∼>80%	a few	[5]
		ass. diverse fauna	no info	∼>80%	a few	
East Pacific Rise	2491–2690	ass. mussel	18–75%	present (po PMA)	no info	[37]
East Pacific Rise	2500	ass. Vestimentifera	2–58%	75–100%	0–25%	[4]
East Pacific Rise	2500	ass. mussel	85±4%	96–97%	3–4%	[39]
Mid Atlantic Ridge	3492	ass. mussel	35±4%	91%	9%	[29]

Location, depth, habitat type (bac mats = bacterial mats; ass. = associated with) and relative abundance of Copepoda within the meiofauna community (% of meio), relative abundance of Dirivultidae within the copepod community (% of cope), and relative abundance of Harpacticoida within the copepod community (% of cope) are given. Reference (ref#) is given for each record. po personal observation.

Apparently, dirivultids are specialized to colonize hard substrate. Their relatively large body with powerful swimming/crawling legs suggests that they are well adapted to an epibenthic life style [26], but they might not be able to live within vent and seep sediments. However, while surfaces of tubeworms, mussels and other foundation species are colonized by dirivultids at vents, they are not inhabited by dirivultids at seeps. Further, whale bones and wood providing large surfaces for colonization are also devoid of dirivultids. We think that the large, continuous area of suitable substrate might play an important role for dirivultids to flourish at midocean ridges, but prevents them from colonizing relatively small patches of hard substrate of biotic origin (e.g. tubes, shells, bones, wood), which are surrounded by soft deep-sea sediments.

The occurrence of dirivultids is restricted to vents and the surrounding axial summit trough, which is in contrast to other meiofauna taxa. Harpacticoid copepod genera found at seeps and vents are usually unknown from deep-sea sediments, but their genera and sometimes even the species are known from shallow water sites (for more details see Martínez Arbizu et al. in prep.). Nematode genera detected at vents and seeps have been reported from deep-sea sediments but also from shallow regions (for more details see Vanreusel et al. in prep).

Dirivultidae were found mostly on hard substrates (basalt and sulfide precipitates) in aggregations of invertebrates, such as bivalves (*Bathymodiolus thermophilus*, *B. puteoserpentis*, *Calyptogena magnifica*), vestimentiferan tubeworms (*Riftia pachytila*, *Ridgeia piscesae*), alvinellids (*Alvinella pompeiana*, *A. caudata*, *Paralvinella sulfincola*, *P. pandorae*, *P. grasslei*, *P. hessleri*), and shrimps (*Rimicaris exoculata*) (Table 5; [4], [5], [9], [11]–[25], [27]–[30]). A total of 24 species each was found within bivalve beds and vestimentiferan bushes. Ten species each were located in alvinellid and shrimp aggregations. Two species were found in bacterial mats growing on basalt, and 3 species were detected in the plankton above vents. Unfortunately, the specific habitat of 8 species (*Aphotopontius baculigerus*, *Fissuricola caritus*, *Rhogobius pressulus*, *Stygiopontius appositus*, *S. brevispina*, *S. lauensis*, *S. verruculatus*) is unknown.

Most dirivultids are habitat generalists as they are able to live at different hydrothermal flux regimes and in different aggregates of megafauna. The majority of species was found in more than 2 different habitats, and only 38% of species were found in a single habitat (6 spp. at bivalves, 5 spp. at shrimps, 4 spp. at vestimentiferans, 1 sp. at alvinellids). 45% of species were detected in 2 habitats, most of them (11 from 19 spp.) in bivalve and in vestimentiferan habitats. 17% (7 spp.) were observed in three habitat types. Since it is known that those megafauna organisms are found at distinct flux regimes (alvinellids and shrimp at high flow with temperatures >50°C, tubeworms at vigorous flow with moderate temperatures (<30°C), bivalves at low flow (<15°C) [31], [32]), most dirivultids must be able to tolerate a wide range of hydrothermal fluid flux regimes.

Information on where exactly and how dirivultids live is rare, since this often requires direct observations. Up to 10 copepods were counted per shrimp (*Rimicaris exoculata*) on the Mid-Atlantic Ridge. They were located on the mouthparts among dense bacteria growth, in the gill chambers, and/or probably were also swimming freely among shrimp swarms [20]. The close-up of a video camera from the submersible showed that dirivultids are crawling on alvinellid tubes colonizing sulfide chimneys at the East Pacific Rise (SG, MB pers. obs.). In this habitat type, temperatures among worms are ranging from 40°C to 100°C, sulfide concentrations can be above 1000 µM and oxygen is depleted [33], [34]. Two of those dirivultid species, *Benthoxynus spiculifer* and *Scotoecetes introrsus* (both found in association with *Paralvinella* spp.), were investigated more in detail and exhibited high hemoglobin concentrations, with a very high and temperature sensitive oxygen affinity. This could be one of the crucial adaptations to live in low-oxygen environments [35], [36].

#### Abundance and diversity

Quantitative data on copepod (and dirivultid) abundances are only available thusfar for the East Pacific Rise (EPR), Juan de Fuca Ridge (JFR), and Mid-Atlantic Ridge (MAR). Copepod abundance at deep-sea hydrothermal vents is on average below 80 ind. 10 cm^−2^, and ranging from 36 to 474 ind. 10 cm^−2^ at alvinellids [5], [25], 1 to 50 ind. 10 cm^−2^ at tubeworms [4], [5], and 13 to 41 ind. 10 cm^−2^ at mussels [29], [37]. They make up 37±23% of total meiofauna communities associated with megafauna aggregations on hard substrates. Dirivultidae are the main copepod family with usually a dominance of 80% (Table 6) [4], [5], [29], [37]–[39].

Interestingly, there are often less males than females in dirivultid populations. For example, the female to male ratio at JFR was 7.6∶1 for *Stygiopontius quadrispinosus*, 10.6∶1 for *Aphotopontius forcipatus*, and 1.5∶1 for *Benthoxynus spiculifer* [5]. Also, many species from tubeworm and mussel associated communities from the Northern EPR showed a female bias or even completely lacked males (*Aphotopontius hydronauticus*, *A. probolus*, *A. acanthinus*). But also, certain species such as *Ceuthocetes acanthothrix*, *C. introversus*, and *Scotoecetes introrsus* were male dominated [39].

In other chemosynthetic habitats no dirivultids have been found and instead harpacticoids were dominant. Similar to vent epifauna, seep epifaunal communities showed a relatively high dominance (34±27%) of copepods within the meiofauna communities. Copepods comprised 10–43% of the meiofauna in tubeworm associated communities, and 17–99% in mussel associated communities [40]. Relative abundance of copepods is lower in sediments from seeps and vents compared to epizooic communities from these habitats. In seep sediments, the relative abundance of copepods was usually <15% within the meiofauna community (Table 6; [41]–[54]). Only 4 samples showed a higher relative abundance [47], [49]. In one sample, in the center of a mud volcano, copepods highly dominated [53], and in another study on bacterial mats the relative abundance of copepods was 33±21% [55]. Vent infauna (most studies are from shallow-water vents) composition is highly variable with relative abundances of copepods ranging from 0 to 68% [38], [56]–[60].

Dirivultid copepod communities are less species rich at high flow alvinellid habitats than at low flow mussel and tubeworm habitats. Copepod communities associated with the alvinellid *Paralvinella sulfincola* at high temperature vents (communities sampled 4 cm away from 255°C peaks) at JFR were highly dominated by *Stygiopontius quadrispinosus* (80%), followed by *Benthoxynus spiculifer* (almost 20%) [5]. A similar dominance pattern was also found at high temperature vents of the EPR, where *S. hispidulus* was the most successful species in alvinellids *Alvinella pompejana* and *A. caudata* habitats [25]. In total 10 species are known from the alvinellid habitat (Table 5).

In contrast, diversity of dirivultids was relatively high at sites with lower temperatures (∼10–20°C). At JFR *B. spiculifer* reached a relative abundance of 60%, and *S. quadrispinosus* of 10%. *Aphotopontius forcipitatus* and various Harpacticoida were additionally present at these lower temperature vents [5]. At the East Pacific Rise, copepod communities associated with the tubeworm *Riftia pachyptila* (max. temp. 18–23°C) and with the mussel *Bathymodiolus thermophilus* (max. temp. 2–10°C) were equally diverse with 6 to 14 copepod species each. Dirivultids dominated the community with 75 to 97%. Most abundant species were *Scotoecetes introrsus* (25±20%), *Benthoxynus tumidiseta* (19±20%), *Ceuthoecetes introversus* (16±13%), *Ceuthoecetes aliger* (13±11%), and *Aphotopontius mammillatus* (12±10%) [39]. A similar copepod diversity pattern was observed in a mussel (*Bathymodiolus puteoserpentis*) associated community at the Mid-Atlantic ridge, where dominant copepods were the dirivultids with *Aphotopontius atlanteus* (57±23%) and *Aphotopontius forcipitatu*s (26±8%). Other copepods included *Halectinosoma* sp. 2 (8±5%), *Aphotopontius temperatus* (4±2%), *Rimipontius mediospinifer* (3±2%) and *Bathylaophonte azorica* (1±1%) [29]. Total number of dirivultid species found at tubeworm and bivalve habitats is 25 and 24, respectively (Table 5).

A conspicuous successional pattern in diversity was found by studying new, mature, and senescent vents at JFR. New vents were mainly colonized by the dirivultid *Aphotopontius forcipitatus* (80%), and mature vents were characterized by a more even distribution of several copepods but with a dominance of dirivultid species. At senescent vents, with no vent flux, dirivultids were low in abundance. These communities were dominated by a cyclopoid species (*Barathricola rimensis*) and various harpacticoid and calanoid copepods [5]. It should be mentioned that there is no information on hydrothermal vent flux temperature from new and mature vents.

#### Trophic interactions

Most dirivultid species can be considered primary consumers and are grazing on bacterial mats and detritus [26], [39]. This could be inferred by analyses of mouthparts and by the finding of partly dissolved bacteria and mucus in the foregut of specimens [26], [38]. Copepods associated with shrimps were feeding on bacteria located on the shrimp mouthparts or on bacteria in the water column [20]. Detailed stable carbon and nitrogen isotopes in combination with fatty acid composition and morphological examination proved that *Stygiopontius quadrispinosus* and *Benthoxynus spiculifer* are mainly bacterivorous and, interestingly, food partitioning at the same trophic level occurred between these two species. *S. quadrispinosus* had a small mouth opening (∼5 µm) and its diet was based on specific bacterial strains, composed of autotrophic bacteria. In contrast, *B. spiculifer* had a larger mouth opening (∼20 µm) and was feeding on various autotrophic and heterotrophic bacteria, [61].

Only members of the genera *Ceuthoecetes*, *Dirivultus*, and *Nilva* have a different form of feeding, and are thought to feed on vestimentiferans [26]. The oral cone of these parasites is cylindrical and the labium is transformed into a cutting borer (Figure 1E). Photographs of vestimentiferans showed round wounds in the tentacular crown which were thought to be inflicted by *Dirivultus dentaneus*. However, it is also stated that indentations could be an artifact caused by the fixation [12]. *Dirivultus spinigulatus* was observed feeding on vestimentiferan plume filaments [21].

Dirivultids are a food source for macrofauna. Stable isotope studies on *Paralvinella* showed that copepods were part of its diet. It was hypothesized that copepods were consumed along with debris while the animal was grazing on the chimney surface [61]. It is unknown yet, but highly probable, that also many other macrofauna species feed on dirivultids.

### Biogeography

**Figure 3:**
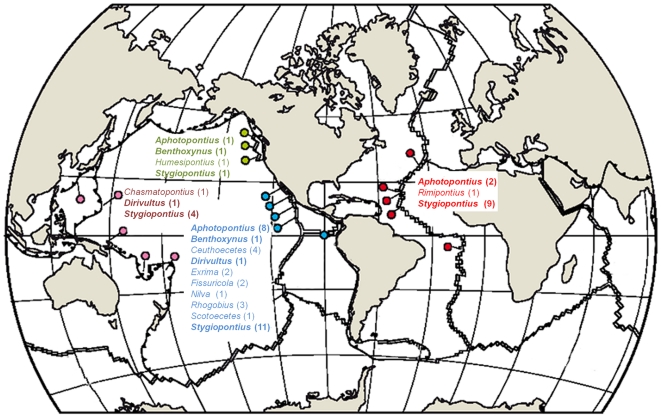
Worldwide distribution of dirivultid genera. Current findings of dirivultid genera on mid-ocean ridges and back-arc basins in the Atlantic (red color code), North East Pacific (green color code), East Pacific (blue color code), and West Pacific (purple color code). The number of species is given between brackets. Map modified after Van Dover et al. [69].

Dirivultids are highly successful in their distribution since they are known from 4 main biogeographic regions, the Atlantic (A), North East Pacific (NEP), East Pacific (EP), and West Pacific (WP) (Figure 3; Table 5; [4], [5], [9], [11]–[25], [27]–[30]). A total of 13 genera with 50 species are currently known and most are endemic to a single region. Only five species occur in 2 regions and those belong to the two most diverse dirivultid genera *Stygiopontius* and *Aphotopontius*. We are not aware of any other region studied, in which dirivultids did occur. It has be taken into account that the majority of studies was historically carried out in the East Pacific. Therefore we expect that future collections will improve our knowledge of the distribution patterns in this family.

The diversity hotspot is the East Pacific with 33 species from 10 genera. Four genera with 4 species are known from the North East Pacific and 3 genera with 6 species from the West Pacific. In the Atlantic, a total of 3 genera with 12 species are currently recognized.

Nine of the 13 genera are endemic. Six genera are restricted to the East Pacific (*Ceuthoecetes* (4 spp.), *Exrima* (2 sp.), *Fissuricola* (1 sp.), *Nilva* (1 sp.), *Rhogobius* (3 spp.), and *Scotoecetes* (1 sp.)). The genus *Chasmatopontius* is only known from the West Pacific (1 sp.), *Humesipontius* only from the North East Pacific (1 sp.), and *Rimipontius* only from the Atlantic (1 spp.) (Figure 3). 45 of the 50 described dirivultid species are endemic to a single a biogeographic region (EP: 30 spp.; A: 8 spp.; WP: 4 spp.; NEP: 3 spp.) (Table 5).

The genus *Stygiopontius* has representatives in all four regions (EP: 11 spp.; A: 9 spp.; WP: 4 spp.; NEP: 1 sp.). *Aphotopontius* was found in the Atlantic (2 spp.), North East Pacific (1 sp.) and East Pacific (8 spp.). *Benthoxynus* is known with a single species each from the North East Pacific and the East Pacific, and *Dirivultus* from the West Pacific (1 sp.) and from off California (*Dirivultus dentaneus*; not at vents) (Figure 3). However, only five species are known from 2 regions. The Atlantic and East Pacific share the species *Stygiopontius mirus* and *S. rimivagus*, the Atlantic and the West Pacific have *S. pectinatus* in common, the Atlantic and North East Pacific *Aphotopontius forcipatus*, and the East Pacific and the West Pacific *S. stabilitus*.

Dispersal of copepods in the pelagial is often but not exclusively during their copepodid stage [6]. Adults and copepodid stages of *Rimipontius mediospinifer*, *Stygiopontius cladarus*, *S. pectinatus* were found in plankton at 80–300 m above vents in the Mid-Atlantic Ridge [28]. Other dirivultids from 9°50′N at the EPR were caught in sediment traps positioned around and above vents (Lauren Mullineaux pers. com., SG pers. obs.). However, copepodids have also been sampled from tubeworm and mussel associations suggesting that at least part of the copepodid development is also possible within the benthos [39]. Although detailed studies on dispersal abilities (such as duration of nauplii and copepodids stages, their buoyancy and feeding strategies) lack, the first observations of copepods and their copepodids in the plankton give a hint that the global distribution of Dirivultidae may have been possible due to long-distance dispersal via ocean currents.

### Origin and phylogenetic relationship

The distribution of extant dirivultids points to a pathway of immigration from shallow waters, and not from the deep-sea sediments, nor from other deep-sea chemosynthetic habitats as it has been suggested for many other vent animals [62], [63]. Dirivultidae are only known from deep-sea hydrothermal vents and from the axial summit trough, but are unknown from deep-sea sediments. We conclude that other deep-sea chemosynthetic habitats did not facilitate immigration as stepping stones towards vents [64] or that dirivultids belong to the wide-spread sulphophilic fauna, because this family is unknown from seeps, whale falls, or any other reducing ecosystems. The only exception is the species *Dirvultus dentaneus*, which was once collected from the siboglinid tubeworm *Lamellibrachia barhami* at 1125 m depth off southern California [12]. *L. barhami* is known from the subduction zone cold seeps on the North America continental margin and from a sedimented hydrothermal region at Middle Valley on the Juan de Fuca Ridge [3]. Due to its limited distribution, it is also unlikely that dirivultids recently originated from a widespread fauna of generalists. Whether dirivultids have a long term *in situ* evolution remains to be tested. For small animals, immigration via their foundation species could be another option to invade the vent habitat. However, we suggest that alvinocarid shrimp, vestimentiferan tubeworms or bivalves did not act as ancestral carrier species. These megafauna species invaded the vent ecosystem via seeps, but dirivultids are not found there [63]. Alvinellid polychaetes are only found at vents, and the order Terebellida (to which alvinellids belong to) is found in shallow waters [63]. We propose that it is most likely that the dirivultid ancestor immigrated from the shallow water, the habitat where nowadays most Siphonostomatoida are found in association with various invertebrates and vertebrates [65]. Maybe, invasion was possible via the hard substrate ecosystem of mid-ocean ridges from shallow waters towards greater depths.

Dirivultidae are considered to have a basal position within the large order Siphonostomatoida due to the presence of an simple oral cone with a loosely associated labrum and labium, instead of a complex oral structure called siphon (with a fused labrum and labium) as found in many other siphonostomatoids [66]. The Siphonostomatoida includes more than 40 families with clear morphological distinction from other copepods (by the formation of an oral cone) but with unresolved phylogenetic relationships [67]. Siphonostomatoids live in association with other animals and most of them are animal parasites exhibiting a siphon for cutting and/or sucking. Two thirds of the species (with a total of >1550) are described as parasites of fishes and mammals, the other third are parasites or associates of invertebrates such as ascidians, polychaetes, bryozoans, cnidarians, crustaceans, echinoderms, or sponges [7]. In contrast, most dirivultids are not parasitic, but are free-living and bacterivorous and often live in aggregations of invertebrates at hydrothermal vents [26], [61]. The bacterivorous feeding type (as seen from the simple mouth structure) of dirivultids suggests that they are basal to the other siphonostomatoids.

The phylogenetic relationships within Dirivultidae are unsolved yet, as detailed morphological comparisons and genetic analyses are by far not complete. The evolution of the formation of the oral cone (a key character of siphonostomatoids) has led to controversial ideas. The first idea, which in our opinion is the most probable one, is that the dirivultid ancestor had a simple oral cone (bacterivorous feeding). This is supported by the bacterivorous species *Chasmatopontius* and *Fissuricola* which are considered basal also due to the existence of 3 inner setae on the distal (second) endopodal segment of leg 4 (see Taxonomy). Over time, Dirivultidae adapted successfully to vents and developed there a more complex oral cone (evolution to a parasitic mode of life). In consequence, the “cutting borer”, a modified distal disk of the oral cone formed by the labium of the parasitic genera *Ceuthoecetes*, *Dirivultus*, and *Nilva* would have evolved secondarily and independent from other parasitic Siphonostomatoida. The second idea is that the feeding apparatus in dirivultids could have evolved from a complex oral cone of secondary consumers (fused labrum and labium) back to a simple oral cone of primary consumers (with a loosely associated labrum and labium). The background of this hypothesis is that other families of the Siphonostomatoida are known to be mostly parasites, and in dirivultids, the antennae, maxillipeds and mandibles have the characteristic form known from those other parasitic Siphonostomatoida [26]. This would imply that *Ceuthoecetes*, *Dirivultus*, and *Nilva* are on the basis of Dirivultidae. However, it should be mentioned here that it remains to be clarified if these morphological features are related to adaptations of the feeding mode (parasitism) or to adaptations of the life style mode of dirivultids (which are free living on foundation species, so antennae could also be used to hold themselves on the foundation species and not to fall off). Interestingly, the Monstrilloida, a former copepod order that was recently placed within the Siphonostomatoida according to molecular analyses, are primary consumers. For this taxon, it has been suggested that they secondarily returned from an ectoparasitic to a free-living mode of life [68]. Only detailed morphological analyses in combination with gene analyses can help unravel the unsolved origin and phylogenetic relationships of Dirivultidae.

### Future perspectives

Dirivultidae is the most diverse taxon at deep-sea hydrothermal vents. With the discovery of new vent sites and with the study of sites where macrofauna species are already known but not the meiofauna, species number is expected to increase further. Although they can be highly abundant in some vent habitats, only a few studies include this family in a broader ecological context. One goal is to take this family into account and the here provided key should help scientists to do so. Biogeographic patterns are expected to change with future collections; especially knowledge from the West Pacific region and the Indian Ocean is very scarce at the moment, and the polar regions remain completely unstudied. Origin and evolutionary processes are unclear yet, and in the future, genetic analyses will help to understand species distributions and speciation processes.
