# Correction: Advances in Taxonomy, Ecology, and Biogeography of Dirivultidae (Copepoda) Associated with Chemosynthetic Environments in the Deep Sea

**DOI:** 10.1371/annotation/d2c17821-286d-4a63-a6ae-348a456d9d0c

**Published:** 2014-01-22

**Authors:** Sabine Gollner, Viatcheslav N. Ivanenko, Pedro Martínez Arbizu, Monika Bright

This article was incorrectly published as a 'Research Article', it is in fact, a 'Collection Review'. Apologies for any inconvenience caused.

